# Epitope-Tagged Autotransporters as Single-Cell Reporters for Gene Expression by a *Salmonella* Typhimurium *wbaP* Mutant

**DOI:** 10.1371/journal.pone.0154828

**Published:** 2016-05-05

**Authors:** Ismeta Curkić, Monika Schütz, Philipp Oberhettinger, Médéric Diard, Manfred Claassen, Dirk Linke, Wolf-Dietrich Hardt

**Affiliations:** 1 Institute of Microbiology, Department of Biology, ETH Zürich, Zürich, Switzerland; 2 Institute of Microbiology and Hygiene, University of Tübingen, Tübingen, Germany; 3 Institute of Molecular Systems Biology, ETH Zürich, Zürich, Switzerland; 4 Department of Biosciences, EVOGENE Section, University of Oslo, Oslo, Norway; Centre National de la Recherche Scientifique, Aix-Marseille Université, FRANCE

## Abstract

Phenotypic diversity is an important trait of bacterial populations and can enhance fitness of the existing genotype in a given environment. To characterize different subpopulations, several studies have analyzed differential gene expression using fluorescent reporters. These studies visualized either single or multiple genes within single cells using different fluorescent proteins. However, variable maturation and folding kinetics of different fluorophores complicate the study of dynamics of gene expression. Here, we present a proof-of-principle study for an alternative gene expression system in a *wbaP* mutant of *Salmonella* Typhimurium (*S*. Tm) lacking the O-sidechain of the lipopolysaccharide. We employed the hemagglutinin (HA)-tagged inverse autotransporter invasin (*invA*_HA_) as a transcriptional reporter for the expression of the type three secretion system 1 (T1) in *S*. Tm. Using a two-reporter approach with GFP and the InvA_HA_ in single cells, we verify that this reporter system can be used for T1 gene expression analysis, at least in strains lacking the O-antigen (*wbaP*), which are permissive for detection of the surface-exposed HA-epitope. When we placed the two reporters *gfp* and *invA*_HA_ under the control of either one or two different promoters of the T1 regulon, we were able to show correlative expression of both reporters. We conclude that the *invA*_HA_ reporter system is a suitable tool to analyze T1gene expression in *S*. Tm and propose its applicability as molecular tool for gene expression studies within single cells.

## Introduction

Most bacteria in the environment live in communities (e.g. biofilms). A characteristic of these communities is the presence of microorganisms with different phenotypes that can take over complementary roles [1]. However, different phenotypes have increasingly been recognized within bacterial cultures that harbor the same genotype and share the same microenvironment. This phenotypic diversity (heterogeneity) within isogenic bacteria has been recognized as common trait among bacterial species and has recently developed into an important research field [2–5]. The functional importance of such phenotypic heterogeneity has remained poorly understood. Phenotypic diversity within isogenic bacterial populations is caused by stochastic (random) events during gene expression, molecular segregation during cell division and/or metabolic activity. In a given system (e.g. a virulence regulatory system), this stochastic behavior, combined with a threshold requirement and a subsequent positive feedback reinforcement that results in a non-linear response can lead to the formation of two (or more) stable phenotypes (= bistable or multistable states (for detailed information on the causes of phenotypic diversity see [6–8]). Phenotypic diversity is thought to have important implications, i.e. in situations of bet hedging and division of labor [9, 10].

In traditional microbial research, bacterial cultures were assumed to be homogenous as they were derived from genetically identical cells. Therefore, outcomes from experiments were usually averaged and researchers completely neglected the influence of different subpopulations in the obtained results. Since the awareness about phenotypic diversity within isogenic bacterial cultures increased, microscopy and flow cytometric analysis in combination with fluorescently labeled molecules of interest, has improved the knowledge of single cell behavior [3, 11–14].

For *Salmonella enterica* subspecies enterica serovar Typhimurium (*S*. Tm), both bet hedging and division of labor have been discussed in the context of the expression of the type three secretion system 1 (T1) virulence factor [15, 16]. *S*. Tm is an enteropathogenic, Gram-negative bacterium that causes self-limiting gastroenteritis [17]. For successful infection, *S*. Tm requires the T1 apparatus, which is encoded on the *Salmonella* pathogenicity island 1 (SPI-1) at centisome 63 of the chromosome [18]. T1 is a key virulence factor for invasion and is expressed in a bistable fashion. Thus, even under inducing conditions *in vitro* and in the host`s intestine only a minority of cells are expressing T1 (T1^+^ cells) [3, 16, 19]. The regulation of T1 has been the subject of numerous studies and the key regulatory factors controlling its expression are well established (for detailed information see [20, 21]). However, the detailed mechanism that leads to bistable expression is still not completely understood. To gain mechanistic insights into the T1 regulation, single cell studies analyzing multiple genes simultaneously would be of interest. Previous studies have used fluorescent protein reporters for analyzing gene expression of one or two genes of interest [2, 4, 22]. Mechanistic analysis would benefit from expanding the number of genes measured in the individual cell. Also, the different maturation and folding properties of different fluorophores have complicated the study of dynamics of gene expression. Thus, new reporter systems with uniform maturation kinetics and the potential to analyze more than two genes per cell would be of significant interest.

Autotransporters are classified as a family of virulence factors that employ a type V secretion system and are capable of transporting their own extracellular passenger domain through the outer membrane. The type V secretion system is comprised of five classes, type Va through to Ve. The Type Ve autotransporter subfamily is characterized by an inverted topology and was only recently described as own class [23–25]. Oberhettinger and colleagues demonstrated the inverted topology of this family using epitope-tagged intimin and invasin [25]. The adhesins intimin and invasin, found in enteropathogenic *E*. *coli* and *Yersinia* species (spp.), respectively, represent two members of the type Ve autotransporter subfamily [26, 27].

Here, we performed a proof-of-concept study to assess if autotransporters may be used as reporters for gene expression in Gram-negative bacteria. In particular, we tested the hemagglutinin (HA)-tagged autotransporter invasin to assess gene expression by *S*. Tm. To establish this system, we chose a two-reporter approach using the fluorophore GFP and the epitope-tagged invasin. Both reporters were placed under the control of promoters present on SPI-1 and thus under the control of the T1 regulon. We demonstrate here that both reporters can be successfully used in one single cell. We show correlative expression of both reporters when driven under the control of one or two different promoters of the T1 regulon. This expands the molecular toolbox for future studies of gene expression within a single bacterial cell.

## Materials and Methods

### Plasmid constructions

A list with all plasmids is provided in Table 1. The suicide plasmid pZ503 harboring *invA*_HA204_ (encoding the invasin gene from *Yersinia enterocolitica* O:8 [25]) downstream of a truncated *sipA* (*sipA; nucleotide 1156–2058 of the open reading frame (orf)) was constructed as follows. The *sipA fragment and *invA*_HA204_ fragments were amplified by PCR from pM1300 and pInvA, respectively, using the primer combinations #52/#53 and #54/#55, respectively. Both fragments were fused by a subsequent amplification step using primers with overlapping restriction sites for *BamH*I (primer #52) and *Not*I (primer #55) and digestion by the restriction endonucleases *BamH*I and *Not*I. The *sipA-invA_HA204_ carrying fragment was ligated into pSB377, which was previously cleaved by the same restriction enzymes, resulting in pZ503.

**Table 1:** Plasmids used in this study.

Plasmid	Characteristics	Resistance	Reference
**pM1300**	*sipA;* pSB377 derivative	tet	[3]
**pM972**	*sicA-gfpmut2;* pBR322 derivative with *gfpmut2* [29] expression under the promoter control of *sicA*	amp	[3]
**pM965**	*rpsM*-*gfpmut2*; pBR322 derivative with constitutive *gfp* expression under the promoter control of *rpsM*	amp	[30]
**pTet-*gfp***	pGM-Tet-GFP; AHTC-inducible expression of *gfp*; high copy	amp	[31]
**pInvA**_**HA**_	pASK-IBA2-*invA*_HA204_; HA-tagged *invA* in pASK-IBA2 vector; AHTC-inducible promoter	amp	[25]
**pZ503**	*sipA-invA_HA204_; pSB377 derivative with InvA_HA_ under the control of the *sicA* promoter which controls the *sicAsipBCDA* operon	tet	This study
**pZ522**	*sipA-gfpmut2-invA_HA204_; pSB377 derivative with GFPmut2 and InvA_HA_ under the control of the *sicA* promoter which controls the *sicAsipBCDA* operon	tet	This study
**pZ523**	*sipA; *sipA* in pGEM-T easy vector (Promega)	amp	This study
**pZ524**	*gfpmut2*; *gfpmut2* in pGEM-T easy vector (Promega)	amp	This study
**pZ526**	*sipA-invA*_HA204_-*gfpmut2;* pZ503 (pSB377) derivative with InvA_HA_ and GFPmut2 under the control of the *sicA* promoter which controls the *sicAsipBCDA* operon	tet	This study
**pZ527**	*sipA-gfpmut2; *sipA-gfpmut2* in pGEM-T easy vector (Promega)	amp	This study
**pZ543**	pASK-IBA2 expression vector; AHTC-inducible promoter	amp	This study
**pASK-IBA2**	Expression vector; AHTC-inducible promoter	amp	[32]
**Int wt**	*eaeA* gene in pASK-IBA2 vector	amp	[32]
**Int wt-Strep**	Int wt with C-terminal Strep-tag in pASK-IBA2 vector	amp	[32]

*truncated *sipA* carrying only nucleotides 1156–2058 of the orf; sm = streptomycin, cm = chloramphenicol, tet = tetracycline, kan = kanamycin, amp = ampicillin

The suicide plasmid pZ526, carrying the *sipA-invA*_HA204_-*gfp* insert, was constructed by cleaving the *gfp* fragment from pM972 using *Not*I and *Sac*II and its ligation into the previously *Not*I- and *Sac*II- digested plasmid pZ503. The suicide plasmid pZ522, harboring the *sipA-gfp-invA*_HA204_ insert, was constructed in three steps, following a modified protocol from An and colleagues [28]. First, the three fragments *sipA, *gfp* and *invA*_HA204_ were amplified using primers with specific overhanging restriction sites to allow directed ligation of all fragments. The amplification of *sipA was done using the primers #124/#125 (overlapping restriction sites *Spe*I and *Afl*II, respectively). The *gfp* fragment was created by amplification with the primers #126/#127 (overlapping restriction sites *AflI*I and *Sal*I, respectively). Finally, *invA*_HA204_ was produced using the primers #128/#129 (overlapping restriction sites *Sal*I and *Sac*II, respectively). The *sipA and *gfp* fragments were each transferred into the pGEM-T easy cloning vector, resulting in pZ523 and pZ524, respectively. In the second step, the *sipA and *gfp* fragments were combined. The *sipA fragment was cut out from pZ523 using the restriction enzymes *Spe*I, *Afl*II. The *gfp* fragment was cut out from pZ524 using *AflI*I and *Sal*I. Both fragments were combined by ligation and subsequent PCR amplification using the primers #124/#127 (*Spe*I and *Sal*I overhangs). The resulting *sipA-gfp containing PCR fragment was introduced into the pGEM-T easy vector (Promega), creating pZ527. In the third step, the *sipA-invA_HA204_ fragment was retrieved from pZ527 by *Spe*I and *Sal*I and ligated with the previously amplified and *Sal*I- and *Sac*II- digested *invA*_HA204_ fragment. After the following PCR amplification step using the primers #124/#129 (harboring *Spe*I and *Sac*II overhangs, respectively), the *sipA-gfp-invA_HA204_ fragment was digested with *Spe*I and *Sac*II and ligated into the pSB377 vector, cut with the same restriction endonucleases. The final ligation step resulted in pZ522. All plasmids were verified by sequencing.

### Bacterial strains

All strains used in this study were derived from *Salmonella* Typhimurium SL1344 [33]. The Tables 2 and 3 list all bacterial strains and primer sequences.

**Table 2:** Strains used in this study.

Strain	Relevant genotype	Derivative of	Resistance	Reference
**SB300**	wt *S*. Tm	SL1344	sm	[33]
**SKI-12**	Δ*wbaP*	SL1344	sm	[36]
**M3142**	*prgH-gfp*+	JH3010, SL1344 [37]	cm	Diard et al., unpublished; *gfp+*: [38]
Z531*	*prgH-gfp*+, sipA-invA_HA204_	M3142	cm, tet	This study
Z536*	*prgH-gfp*+, *sipA-invA*_HA204_ *wbaP*::*aphT*	Z531	cm, tet, kan	This study
Z532*	*sipA-invA*_HA204_	χ8602, SL1344 [34]	sm, tet	This study
Z537*	*sipA-invA*_HA204_ *wbaP*::*aphT*	Z532	sm, tet, kan	This study
Z555*	*prgH-gfp*+ *wbaP*::*aphT*	M3142	sm, kan, cm	This study
**Z562**	*sipA-invA*_HA204_-*gfpmut2*	SB300	sm, tet	This study; *gfpmut2*: [29]
Z565*	*sipA-invA*_HA204_-*gfpmut2*	X8602	sm, tet	This study
Z567*	*sipA-invA*_HA204_-*gfpmut2 wbaP*::*cat*	Z565	sm, tet, cm	This study
**Z569**	*sipA*-*gfpmut2*-*invA*_HA204_	SB300	sm, tet	This study
Z572*	*sipA-gfpmut2-invA*_HA204_	X8602	sm, tet	This study
Z574*	*sipA-gfpmut2-invA*_HA204_ *wbaP*::*cat*	Z572	sm, tet, cm	This study

* Unflagellated non-motile χ8602 background (Δ*fliC*, Δ*fljB*); wt = wild type

**Table 3:** Primer sequences used in this study.

#	Primer	Primer sequence 5`-3`	Purpose
52	SipA-BamHI-fw	GATGACGGATCCATTGACCATGGCATCGCGGG	1)
53	SipA-InvA-rv	CACAGTTAGCGTATTAAAAAATGAATACATTGAATTCCTCCTCTAACGCTGCATGTGCAAGCCATC	1)
54	InvA-fw	ATGTATTCATTTTTTAATACGCTAACTG	1)
55	InvA-NotI-rv	GACGATGCGGCCGCCTATTGAGGCTCCGCACACAG	1)
10	_hilEM45-ctrl-Fw2	GACAACAAGCCAGGGATGTAAC	3)
11	_hilEM45-ctrl-Rv2	GGGAGTAAACAGGAGACAAGTG	3)
60	SipA-seq	TATCGGCAAGCCGGTACAGG	3)
61	SipA-seq2	GGCGTGGATCGGGTTATTAC	3)
48	InvA-seq4	CGCTGGAAGCGGAGACTATAATG	3)
42	InvA-seq2	GGCGGTCAATACCTATACCC	3)
43	InvA-seq3	CCGGCATTAACGTGAATGGTG	3)
68	SipA-ko-ctrl-fw	CGTTGATCTGACGCCATTAC	2)
70	InvA-SipA-rv	TAATGTTGCTGTGAGAACCCATA	2)
71	InvA-SipA-fw	TCTAATCCAGCTACGTTGAC	2)
69	SipA-ko-ctrl-rv	GAGTCAGCGTAAAGATCCTC	2)
62	wbaP-pkd-fw	CTTAATATGCCTATTTTATTTACATTATGCACGGTCAGAGGGTGAGGATTAAGTGTAGGCTGGAGCTGCTTC	1)
63	wbaP-pkd-rv	GATTTTACGCAGGCTAATTTATACAATTATTATTCAGTACTTCTCGGTAAGCCATATGAATATCCTCCTTAGTTCCTATTCC	1)
64	wbaP-ko-ctrl-fw	CCAACTCGTTACACCCATTC	2)
65	wbaP-ko-ctrl-rv	TCGATAGCTGCATCAGTACC	2)
66	aphT-ctrl-rv	CGGGTAGCCAACGCTATGTC	2)
67	aphT-ctrl-fw	CCGGTGCCCTGAATGAACTG	2)
124	_sipA-SpeI-fw	GATGACACTAGTATTGACCATGGCATCGCGG	1)
125	_sipA-AflII-rv	GACGATCTTAAGCTAACGCTGCATGTGCAAG	1)
126	GFP-AflII-fw	GATGACCTTAAGCTGCAGGAATTCAGGAGGT	1)
127	GFP-SalI-rv2	GACGATGTCGACTTATTTGTATAGTTCATCC	1)
128	InvA-SalI-fw	GATGACGTCGACAGGAGGAATTCAATGTATTCATTT	1)
129	InvA-SacII-rv	GACGATCCGCGGCTATTGAGGCTCCGCACACAG	1)
144	sipA-seq-rv	CGCTGCATGTGCAAGCCATCAAC	3)
145	InvA-seq-rv	ACGGTCAACGTAGCTGGATTAG	3)

Purpose:

^1)^ strain/plasmid construction,

^2)^ PCR verification,

^3)^ sequencing

The suicide plasmid pZ503 was introduced into M3142 and the unflagellated non-motile strain χ8602 [34] by conjugation, thereby creating Z531 and Z532, respectively. In the strains Z531, Z532 and M3142, the *wbaP* gene was deleted as previously described [35]. In short, the primers #62 and #63, harboring the λ recombinase recognition sites and sequences adjacent to the *wbaP* gene, were used to amplify the kanamycin resistance cassette from pKD4. The PCR product was electroporated into Z531, Z532 and M3142, each harboring pKD46 to create an in-frame deletion of *wbaP* directly in the desired strains. The pKD46 plasmid was directly transformed into the strains of interest to avoid the need for an additional P22 phage transduction step (note: P22 phage binds to the O-antigen, which is deleted in these strains). Thereby, the final strains Z536, Z537 and Z555, respectively, were constructed. The presence of the insert was verified by PCR using the primers #68/#70. The primer combinations #64/#65 and #64/#66, respectively, were used to confirm the *wbaP* deletion.

The suicide plasmids pZ526 and pZ522, carrying the *sipA-invA*_HA204_-*gfp* and *sipA-gfp-invA*_HA204_ inserts, respectively, were each individually integrated into the chromosome of SB300. This created the strains Z562 and Z569, respectively. Subsequently, P22 phage transduction was performed to introduce the inserts of pZ526 and pZ522 individually into the χ8602 background, resulting in Z565 and Z572, respectively. To obtain Z567 and Z574, the *wbaP* gene was replaced by a chloramphenicol resistance gene cassette by amplification of the resistance cassette from pKD3, as described before. The integration of the two inserts was verified by PCR using the primers #68/#70. For confirming the *wbaP* exchange by the chloramphenicol cassette, the primer combination #64 and #65 was used.

### Culturing of bacterial strains

Overnight cultures were diluted 1:20, except when stated otherwise, in fresh LB broth (0.1 M NaCl) and grown for 4 h at 37°C, before aliquots for Western blot analysis were taken and subsequent staining was performed. For the growth-curve experiments, overnight cultures were diluted 1:100 and the optical density (OD_600_) was measured at the indicated time points. In all induction experiments, we either used anhydrotetracycline (AHTC, Chemie Brunschwig AG) or L-arabinose at the stated concentrations. Except otherwise stated, the induction was done immediately after dilution of the overnight culture.

### Immunofluorescence measurements

Subcultures were grown for 4 h as described before. 10 μl of the subculture containing approximately 1x10^7^ cells, were transferred onto V-shaped 96-well plates (Nunc 96-well polypropylene MicroWell Plates, Thermo Scientific). Before staining, cells were washed 2x with 200 μl washing buffer (PBS, 4% sucrose, 0.02% sodium azide) on the plate by centrifuging at 4°C at 4000 rpm for 10 min. Blocking was done with 200 μl blocking buffer (washing buffer containing 3% bovine serum albumin (BSA)) for 1 h at 4°C, before cells were stained in 25 μl blocking buffer containing first the monoclonal mouse anti-HA (1:1000, clone HA-7, Sigma) and then the secondary goat anti-mouse-Cy5 antibody (1:200, Jackson ImmunoResearch) for 1 h, 4°C. Washing between and after the staining steps was done with PBS as described above. Finally, the cells were resuspended in 200 μl PBS and surface staining of the HA tag was analyzed by flow cytometry and fluorescence microscopy.

Flow cytometric analysis was done using an LSR II analyzer (BD Biosciences) by measuring forward and sideward scatter of bacterial cells in addition to GFP, representing the respective transcriptional fusions, and the surface-stained HA tag (GFP and Alexa Fluor 647 laser, respectively). The data was analyzed with FlowJo software (version 10.0.8).

For microscopy, 150 μl of the previously stained cells were transferred onto gelatin (0.2%) coated cover slips. Cover slips were prepared as previously described [39]. The cover slips harboring stained bacterial cells were mounted onto glass slides using Mowiol and kept in the dark at RT overnight before placing them at 4°C to store for further analysis. The fixed slides were analyzed the next day using an Axiovert 200m microscope with a spinning disc confocal laser unit (Visitron) and a solid state laser unit (Toptica). The samples were analyzed using the lasers for either GFP (488 nm) or Cy5 (647 nm) excitation. Data analysis was done using Volocity 6.3 and GraphPad Prism (version 6.07). The correlation analysis was performed using the Spearman correlation analysis in GraphPad Prism. Spearman rank correlation is a non-parametric test to measure the degree of association between two variables. In contrast to linear regression analysis, this test does not make assumptions about the distribution of the samples or the dependency of the analyzed parameters and is used for ordinal or nominal data.

**Fig 1:**
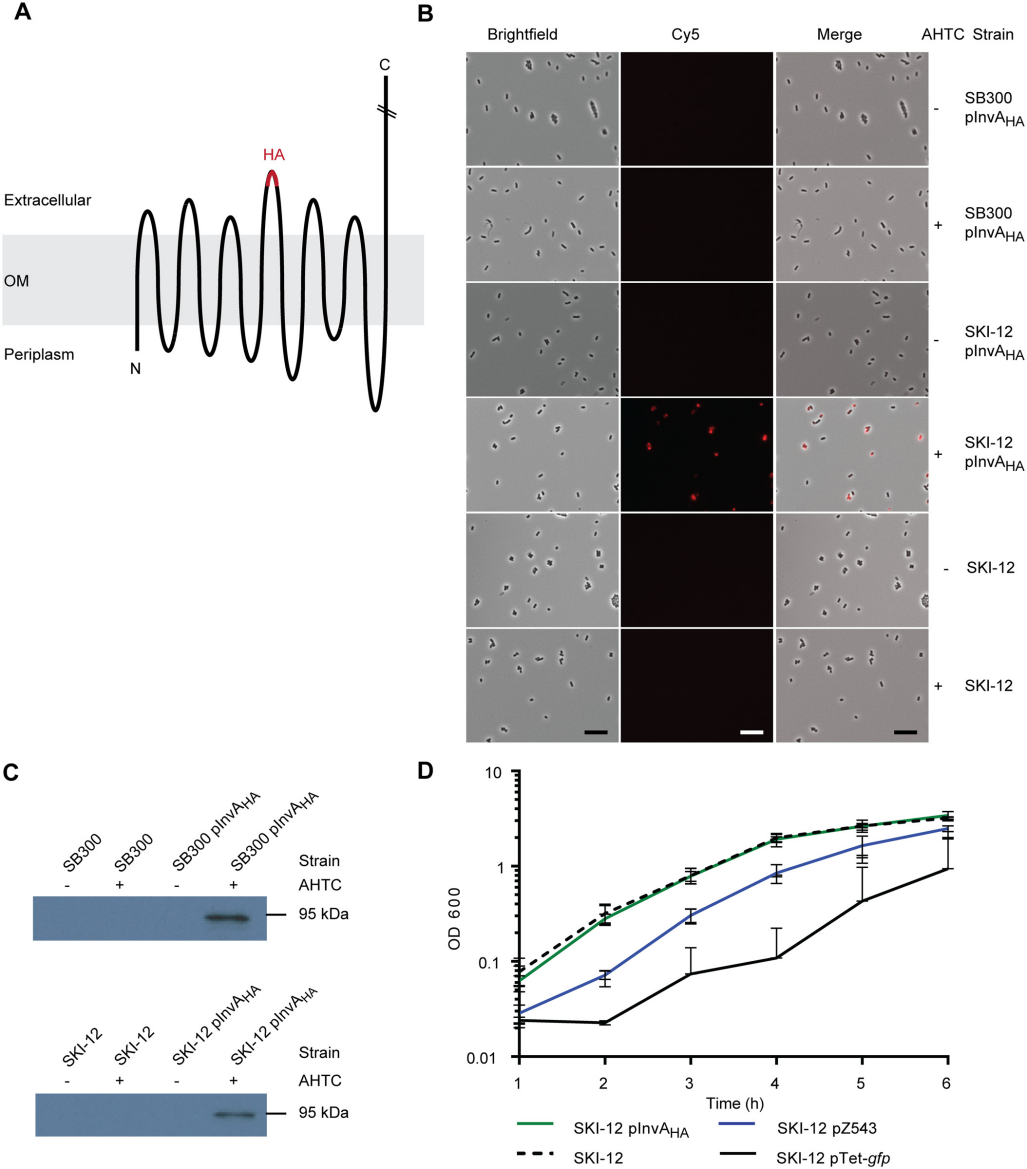
Scheme of HA epitope topology in invasin and characterization of plasmid based InvA_HA_ expression. **A)** Schematic drawing of the HA epitope tag position within the β-barrel of invasin. Six extracellular loops extrude to the extracellular space, with loop 4 displaying the HA epitope tag. The C-terminal part of the passenger domain reaches out into extracellular space. Figure adapted from [25]. **B)** Microscopic analysis of HA-specific staining in SB300 and SKI-12 (Δ*wbaP;* O-antigen-deficient strain, [36]). The indicated strains harboring the pInvA_HA_ plasmid were stained using the quick staining protocol described in the material and methods section. The scale bar represents 10 μm. **C)** Western blot analysis of HA protein levels upon induction of expression in SB300 and the O-antigen-deficient SKI-12. InvA_HA_ size ~94 kDa. **D)** Growth curves upon induction with 200 ng/ml AHTC. pZ543 is the "empty" control vector and lacks *invA*_HA_. pTet-*gfp* is a control plasmid expressing GFP from the tetracycline promoter. Mean and standard deviation are plotted. OM = outer membrane; +/- indicates presence or absence of 200 ng/ml AHTC.

For Fig 1B, a quick staining protocol was applied. In short, bacteria from 1.5 ml of the 4 h subculture were pelleted and washed in blocking buffer (PBS containing 5% BSA). Blocking was done in 1 ml volumes for 1 h at 4°C, before the mouse anti-HA (1:5000, clone 12CA5, Roche) and goat anti-mouse-Cy5 (1:200, Jackson ImmunoResearch) antibodies were added. Antibody incubation was done at 4°C for 1 h. Cells were then transferred onto agarose pads (1% agarose in PBS) and analyzed using the Zeiss Axioplan 2 microscope (Zeiss) using the same software as described above.

### Western blot analysis

Whole cell lysates of bacteria were prepared by resuspending bacterial pellets in Laemmli Sample Buffer to obtain 1x10^9^ bacteria/ml, before incubation at 95°C for 5 min. The proteins were resolved by SDS-PAGE and subsequently transferred onto nitrocellulose membranes. Subsequently, the membranes were Coomassie-stained to obtain protein loading controls. In short, membranes were washed 3x for 2 min in ddH_2_O, before they were stained with the filter stain (Coomassie brilliant blue R 0.05% final concentration). Membranes were de-stained for 5 min with the de-stain solution to allow protein visualization and dried at RT. For the antibody staining, the membranes were blocked overnight at 4°C or 1 h at room temperature (RT) in PBS/T (PBS, 0.1% Tween20) containing 5% milk powder. The blots were stained with monoclonal mouse anti-HA (1:1000, clone HA-7, Sigma), monoclonal mouse anti-GFP (1:2000, clone 7.1 and 13.1, Roche) and secondary peroxidase-conjugated goat anti-mouse antibody (1:4000, Sigma). The Page Ruler Plus Prestained Protein Ladder (10–250 kDa, Thermo Scientific) was used as molecular size marker.

## Results

### Analysis of a plasmid-based *invA*_HA_ expression construct

An HA-tagged invasin (*invA*_HA_) reporter plasmid had previously been designed for topological studies of the type Ve autotransporters in *E*. *coli* [25]. This previous work had verified that the invasin gene from *Yersinia enterocolitica* O:8 can efficiently be displayed on the surface of other bacterial species. The *invA*_HA_ fusion gene harbored a double HA tag linked by the amino acid triplet GSG (GSG-HA-GSG-HA-GSG) within loop 4 of the invasin`s β-barrel at the position A204. The double HA tag is referred to as HA tag throughout the paper and its location is illustrated in Fig 1A. The fusion gene was located on a pASK-IBA2 plasmid backbone and its expression was controlled by a tetracycline-regulated promoter. This promoter can be induced using anhydrotetracycline (AHTC), a tetracycline derivative, which lacks antibiotic activity. To verify the expression of the HA epitope tag in *Salmonella* spp., we transformed the *invA*_HA_-harboring plasmid into the SL1344 *S*. Tm derivative SB300 [33]. However, while Western blots of bacterial lysates verified the expression of InvA_HA_ in SB300, the staining of the intact bacteria with anti-HA antibodies did not yield any HA-specific signal (Fig 1B and 1C). As the HA tag was located at the extracellular loop of the invasin β-barrel, we reasoned that the absence of anti-HA antibody staining might be attributable to shielding by the lipopolysaccharide (LPS) layer that prevented antibody access to the HA epitope.

We therefore decided to test for HA staining using *S*. Tm SKI-12, an isogenic mutant that was lacking the O-antigen polysaccharide moiety of LPS. The expression of the O-antigen was ablated due to the deletion of *wbaP*, which encodes a phosphogalactosyltransferase, essential for the initiation of the O-antigen biosynthesis [36]. The earlier work had demonstrated that this mutation can be complemented. As expected, SKI-12 pInvA_HA_ expressed the InvA_HA_ protein upon induction with AHTC, as shown by surface staining of induced bacterial cells and Western blot (Fig 1B and 1C). In conclusion, these data suggested that the O-antigen layer of the SB300 strain prevented antibody staining of the β-barrel-positioned HA epitope of invasin. Therefore, we have chosen SKI-12 as the background strain for the following experiments.

The expression of some proteins, including the expression of the T1 apparatus or even GFP can impose a significant burden upon the bacterial cell [39, 40]. This can affect bacterial physiology as indicated by reduced growth rates or reduced colonization. To investigate whether the expression of the *invA*_HA_ reporter had an influence on the growth of the bacterial cells, we performed a growth experiment and measured the growth of the cells upon induction of InvA_HA_ expression. As shown in Fig 1D, the induction of InvA_HA_ in the SKI-12 background did not significantly affect growth. SKI-12 harboring an empty control vector served as an additional control. In contrast, a tetracycline-inducible *gfp* expressing plasmid, which was known to inflict a burden to *S*. Tm, showed a much lower growth rate. In addition, we tested the effect of gradually increasing the expression of the *invA*_HA_ reporter. In line with the data shown above, gradual induction increased the reporter surface staining on SKI-12 (S1 Fig). This suggested that the *invA*_HA_ reporter can be used to monitor gene expression in SKI-12 and that reporter expression can be monitored over a wide range of induction levels.

### Deletion of *wbaP* has a slight effect on T1 expression

**Fig 2:**
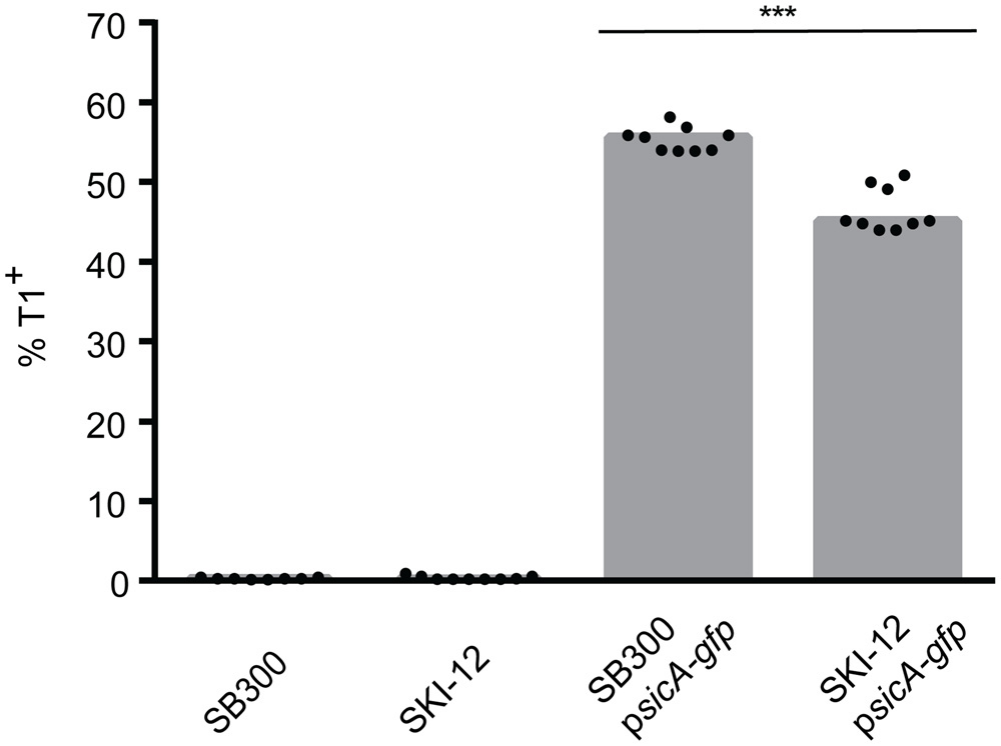
Effect of *wbaP* deletion on T1 expression. SB300 or SKI-12 (Δ*wbaP*) were analyzed for T1 expression using the T1 reporter plasmid p*sicA-gfp* (pM972), [3]. T1 expression was assessed by flow cytometric analysis. The data were derived from three independent experiments. Statistical significance was assessed using the Mann-Whitney test.

In order to probe the suitability of *invA*_HA_ as a transcriptional reporter, we wanted to compare it to the well-established *gfp* reporter. In particular, we wanted to assess SPI-1 expression. In the past, the expression of the T1 was mostly analyzed in the *S*. Tm background with O-antigen-proficient LPS (smooth LPS, SB300) [3, 19]. In order to use the T1-*gfp* reporter in an O-antigen-deficient *wbaP* background, we first had to establish SPI-1 expression and monitor T1-*gfp* in this strain. To this end, we used the plasmid-based T1 reporter, expressing *gfp* under the promoter control of the SPI-1-encoded chaperone SicA [3]. The *sicA* promoter controls the expression of the operon, encoding *sicAsipBCDA* [20]. Flow cytometric analysis revealed that T1 expression was reduced (from ~ 55% to ~ 45%) in the absence of the O-antigen polysaccharide Fig 2). Nevertheless, T1 is expressed at appreciable levels. Therefore, the Δ*wbaP* strain could be used for our proof-of-concept work to test if autotransporters can be used in principle as transcriptional gene expression reporters. The use of the Δ*wbaP* strain of InvA_HA_ was of advantage, as the HA epitope could be stained in the O-antigen-deficient strain, in contrast to the SB300 strain harboring a smooth LPS layer (Fig 1B).

### Chromosomal *invA*_HA_ reporters for the expression of the SPI-1 promoters P*prgH* and P*sicA*

To assess the suitability of the autotransporter approach, we generated strains carrying the *invA*_HA_ reporter in the chromosome. For this purpose, we focused on T1. First, we intended to verify that the T1 expression displayed by the *invA*_HA_ reporter is comparable to the *gfp*-based T1 reporter system used in previous studies [3, 15, 19, 37]. We therefore used the previously published *prgH* promoter-driven *gfp* (P*prgH-gfp*) reporter strain JH3010 used by the Hinton laboratory to visualize T1 expression [37]. PrgH is a structural protein of the T1 needle and is encoded on one of the three large operons of SPI-1. We constructed all strains in a non-motile phenotype [34] to allow future analysis of these strains in single cell setups, such as the microfluidic setup [41]. By P22 transduction we introduced the P*prgH-gfp* allele of the JH3010 strain into the flagella-mutated background strain (χ8602), before we introduced the *invA*_HA_ reporter downstream of the SPI-1 encoded effector *sipA* [42]. SipA is the last gene of the *sicAsipBCDA* operon and expressed under the control of the *sicA* promoter. Hence, both reporters, P*prgH*-driven *gfp* and P*sicA*-driven *invA*_HA_, were under the control of the T1 regulon and should be expressed by the same cells. Finally, to allow HA-specific staining, we introduced *wbaP* deletions into all strains used for the following experiments (see Table 1).

**Fig 3:**
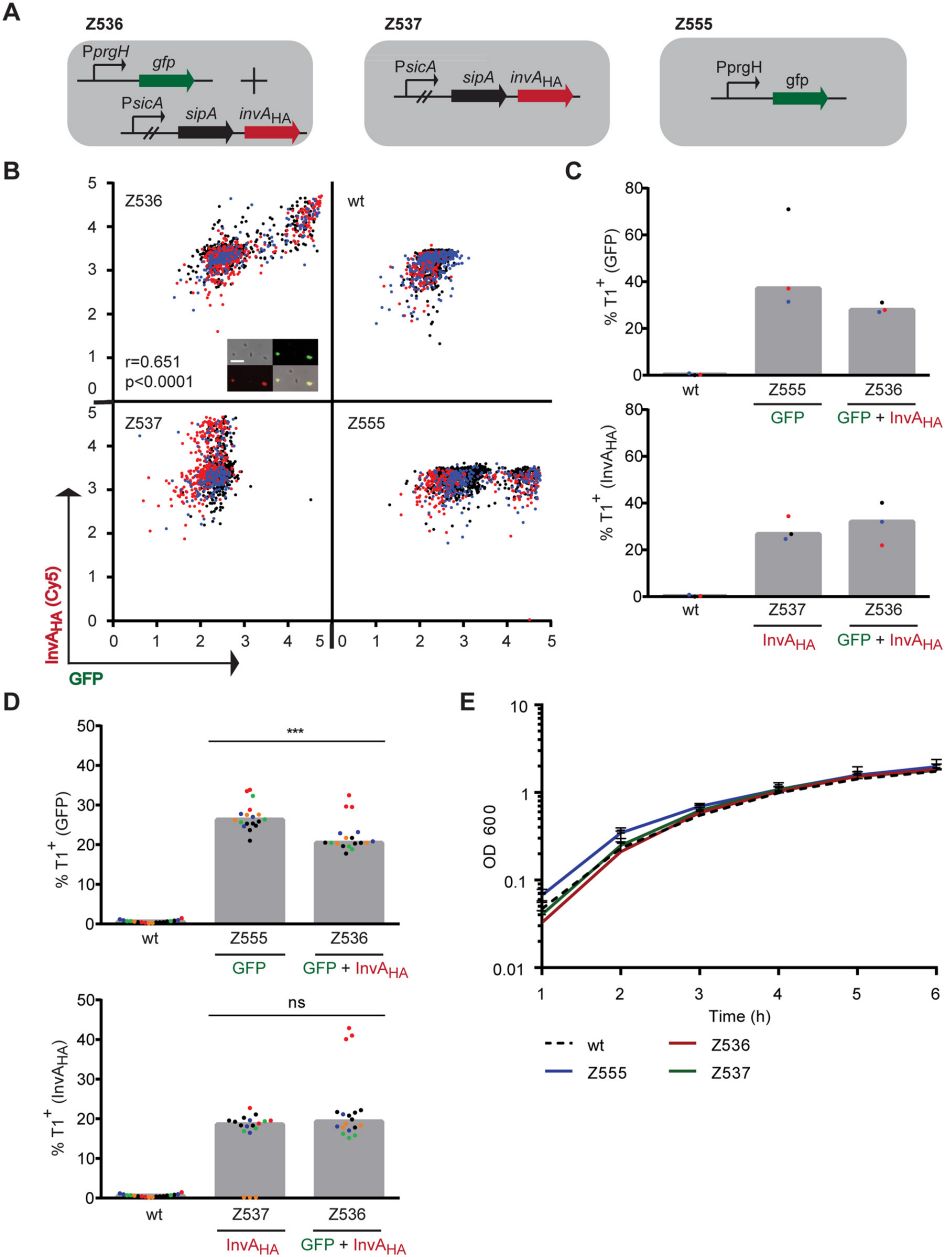
Scheme of chromosomal constructs of Z536, Z537 and Z555, expressing the *gfp* and *invA*_HA_ reporters under the control of two SPI-1 encoded promoters and analysis of the constructs. **A)** Schematic drawing of the chromosomal transcriptional fusions in the indicated strains. The reporters were under the control of either the *prgH* promoter (P*prgH*; Z555) or the *sicA* promoter (P*sicA*; Z537) or both (Z536). The cells were stained as described in the material methods section. We used cells from the same preparation for either microscopic **(B, C)** or flow cytometric **(D)** analysis. Triplicates were measured by flow cytometry **(D),** whereas these samples were combined for microscopic analysis **(B, C). B)** Analysis of *gfp* and *invA*_HA_ expression of the indicated strains, as measured by microscopy. The *invA*_HA_ staining was measured by the Cy5 exciting laser for microscopy. The GFP and Cy5 fluorescence values of all individual cells were extracted and the log10 transformed values were plotted against each other. The three colors indicate the different independent experiments. Correlation was analyzed by Spearman correlation analysis (see material and methods for detailed information). Fluorescence of GFP and InvA_HA_ (Cy5) is indicated in arbitrary units (AU). Insert: Microscopy images from the original data set. This illustrates the staining strategy and the nature of the primary data used for the expression analysis. Images represent the phase contrast, GFP (green) and Cy5 (red; HA epitope) channels and the merge (orange; from left top to right bottom). White bar: 5 μm. **C)** Quantitative analysis of the data set from B. The upper and lower graphs display the percentages of cells displaying GFP or Cy5 (for InvA_HA_) fluorescence, respectively. The thresholds for GFP and Cy5 (for InvA_HA_) fluorescence were set by the maximum values in the GFP or Cy5 channel of the wt strain, respectively. Z555 and Z537 are the controls, harboring only the *gfp* or the *invA*_HA_ reporter, respectively. Z536 harbors both reporters, *gfp* and *invA*_HA_. **D)** Flow cytometric analysis of the same cells as in B). The upper and lower graphs display the percentages of T1^+^ cells, measured by the GFP and Alexa Fluor 647 (for InvA_HA_) excitations. Statistical significance was assessed using the Mann-Whitney test. **E)** Growth analysis of the chromosomally tagged strains. Mean and standard deviation of three independent experiments are shown. "wt" = wild type SKI-12. ns = not significant; *** p<0.0002.

In this way, we obtained a non-motile strain in an O-antigen-deficient background (*wbaP* deletion), harboring P*prgH-*driven *gfp* and Ps*icA*-driven *invA*_HA_ expression (Z536, Fig 3A). Furthermore, we constructed two control strains (Z537 and Z555, respectively; Fig 3A), harboring either the P*sicA*-driven *invA*_HA_ or the P*prgH*-driven *gfp* reporter. These latter strains should allow us to control for potential effects caused by the expression of two reporters within the same cell. The cells were grown in LB medium and bacteria were stained for the HA epitope tag. The GFP levels could be measured without any additional staining. Analysis of the stained cells was done by microscopy and by flow cytometry. Single cell expression analysis of Z536 verified that the *gfp* and *invA*_HA_ reporters are indeed co-expressed (r = 0.651, p<0.0001). As expected, the control strains Z537 and Z555 displayed either the InvA_HA_ or the GFP-specific signal, respectively (Fig 3B). Quantification of the GFP signal revealed that the expression of both reporters within the same cell (i.e. in Z536) was virtually equivalent to that observed in the strains expressing just one reporter at a time (i.e. Z537 and Z555). Only the P*prgH-gfp* reporter showed a slight reduction in strain Z536 (Fig 3C).

To obtain further evidence, we also performed FACS analysis of the same samples that had been used for microscopy. Again, we observed strong co-expression of P*prgH-gfp* and of P*sicA-invA*_HA_ and a slight reduction of P*prgH-gfp* expression in Z536 (compare to Z555, Fig 3D). This indicated that the *invA*_HA_ reporter is indeed suitable for monitoring T1 expression.

Next, we analyzed whether the integration of the different reporters resulted in negative effects on the growth of cells at the population level. We did not observe any adverse effects on growth of the new reporter strains (Fig 3E). However, as only a subpopulation of cells expresses T1 [3], the absence of a growth phenotype in this bulk analysis does not reflect the single cell situation of cells harboring the newly introduced reporters. Single cell analysis e.g. by using agar pads [3] or by using microfluidics [15, 41] may help to further back this up.

### Growth phase-dependent T1 expression is detected by the *gfp* and the *invA*_HA_ reporter.

**Fig 4:**
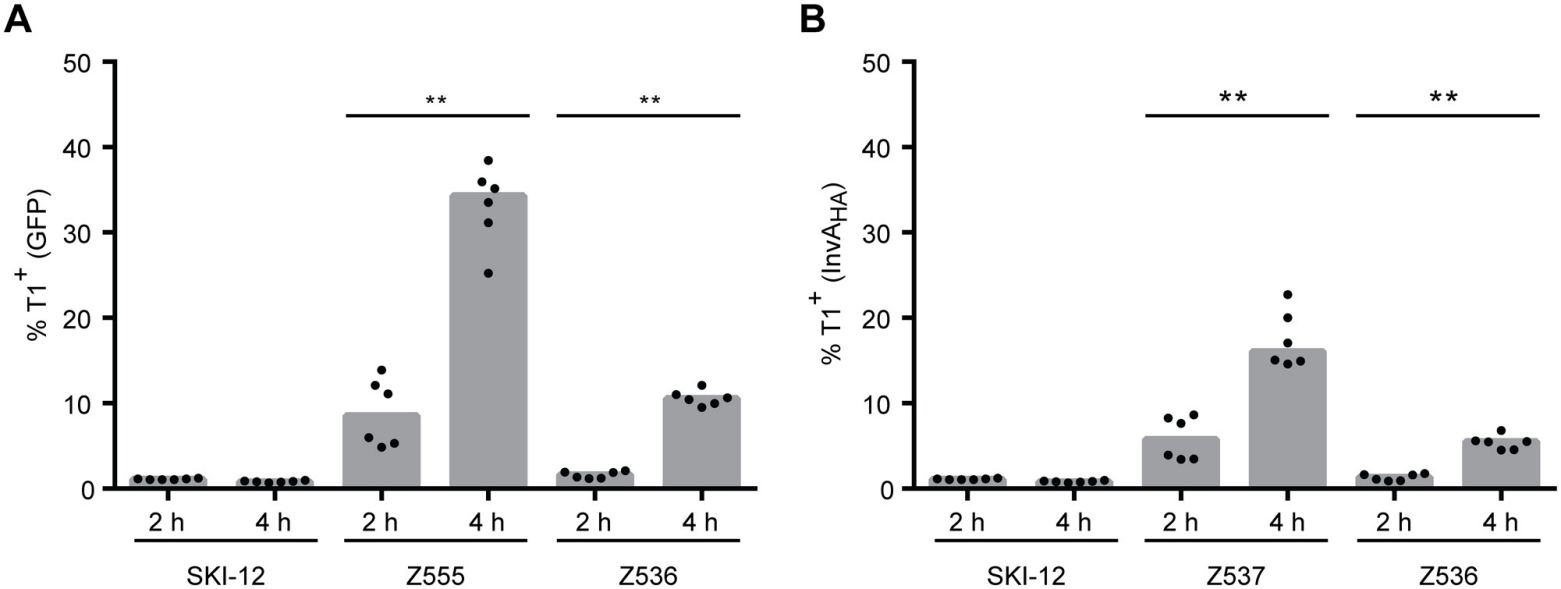
GFP and InvA_HA_ expression during different growth phases. Flow cytometric analysis of *gfp* **(A)** and *invA*_HA_ expression **(B)** of the indicated strains, measured by GFP and Alexa Fluor 647 (for InvA_HA_) excitations, respectively. Z555 and Z537 are the controls, harboring only the *gfp* or the *invA*_HA_ reporter, respectively. Z536 harbors both reporters, *gfp* and *invA*_HA_. The subcultures were diluted 1:400 prior to incubation for 2 or 4 h, respectively. Statistical significance was tested using the Mann-Whitney test. ** p = 0.0022.

To test the performance of the *invA*_HA_ reporter, we analyzed the well-established effect of the growth phase on T1 expression at the single cell level [3]. Thus, cultures of the reporter strain were inoculated from overnight cultures (1:400; this avoids spillover T1 expression that can occur in the overnight culture) and cultures were grown for 2 h (non-inducing condition) or for 4 h (T1 induction). Then, the HA epitope was stained and GFP and HA-staining were analyzed by flow cytometry. As expected, the reporter-less control strain SKI-12 did not show any fluorescence signal, while Z555 and Z536 expressed higher GFP levels at 4 h than at 2 h (Fig 4A). Equivalent results were obtained for the *invA*_HA_ reporter expression by Z537 and Z536 (Fig 4B). In conclusion, these experiments showed that *invA*_HA_ reporters are suitable for monitoring gene expression and suggested that this construct can be combined with established *gfp* reporters to monitor two different promoters in the same bacterial cell.

### Expression of the *gfp* and *invA*_HA_ reporters under the control of the same promoter

**Fig 5:**
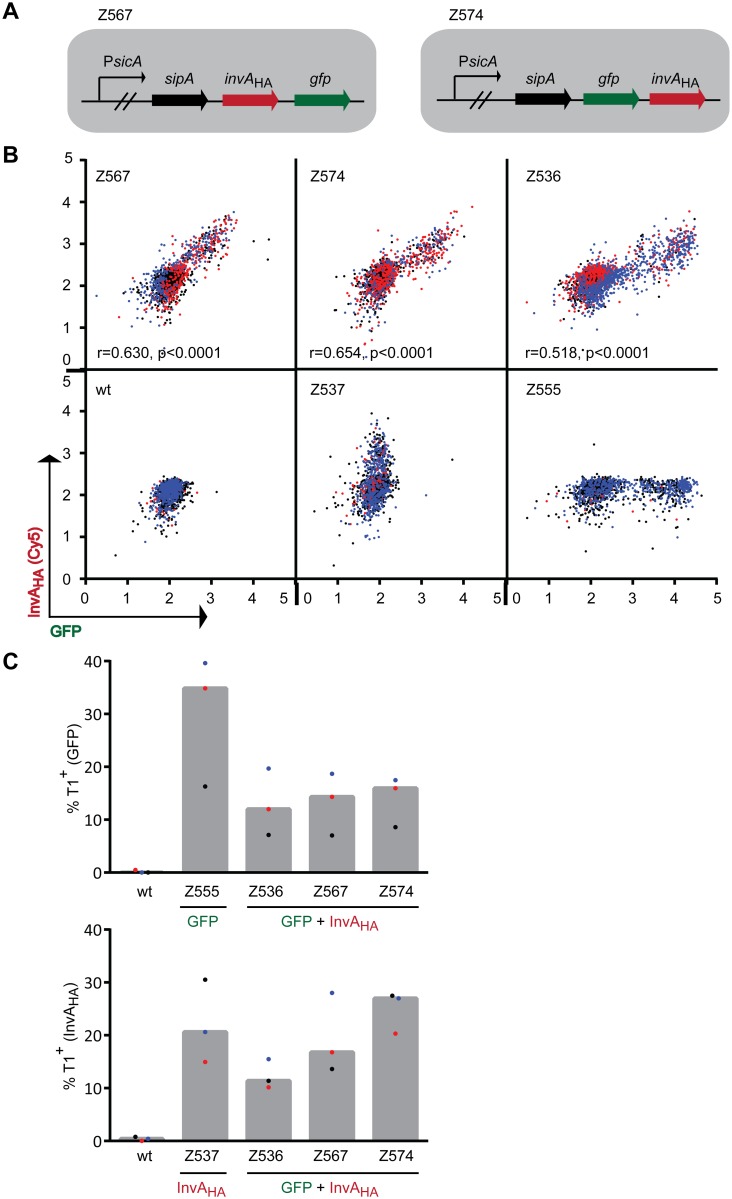
Expression of the two reporters *gfp* and *invA*_HA_ under the control of the same P*sicA* promoter. **A)** Scheme of the transcriptional fusions of the *gfp* and *invA*_HA_ reporters, which were inserted downstream of *sipA*. **B)** Microscopic analysis of GFP and InvA_HA_ fluorescence levels (measured by the Cy5 exciting laser for microscopy) of the indicated strains. The three colors indicate the data from different experiments. The correlation was analyzed by Spearman correlation analysis. Fluorescence of GFP and InvA_HA_ (Cy5) is indicated in arbitrary units (AU). **C)** Quantitative analysis of the data set from B. The upper and lower graph display the percentages of cells expressing GFP or Cy5 (for InvA_HA_) fluorescence, respectively. The thresholds for GFP and Cy5 (for InvA_HA_) fluorescence were set using the maximum values in the GFP or Cy5 channel of the wt untagged strain, respectively. Z555 and Z537 were used as controls, harboring only the *gfp* or the *invA*_HA_ reporter, respectively. Z536 harbors both reporters (*gfp* or the *invA*_HA_) under the control of two different SPI-1 promoters. Z567 and Z574 harbor both reporters under *sicA* promoter control. **D)** Western blot analysis of GFP and HA protein levels in the indicated strains. p*rpsM-gfp* (pM965, [30]) served as a positive control for GFP expression. GFP size = ~27 kDa, InvA_HA_ size ~94 kDa. The Coomassie stained membrane serves as loading control. "wt" = SKI-12.

So far, we have demonstrated that the use of the P*sicA*-driven *invA*_HA_ reporter can monitor T1 expression. However, the correlation with the P*prgH-gfp* reporter was below 100%. It had remained unclear if slight levels of divergence might arise from the different folding/maturation kinetics of the two reporters, the noisiness in promoter expression or to other so far unidentified effects. In a first attempt to address this, we wanted to test whether the combination of both reporters under the control of the same promoter will result in a similar pattern of correlation. For this purpose, we generated a transcriptional fusion of both constructs downstream of *sipA*, thus placing both constructs under the control of the *sicA* promoter (Fig 5A). To control for any artefacts caused by the arrangement of reporters, we generated two strains, one carrying the *gfp* downstream of the *invA*_HA_- and one carrying *invA*_HA_ downstream of the *gfp* reporter. This yielded the two strains harboring either *sipA-invA*_HA_-*gfp* (Z567) or *sipA-gfp-invA*_HA_ (Z574; Fig 5A). Single cell expression analysis revealed that both reporters are expressed and that their expression was correlated (Z567: r = 0.630, p<0.0001; Z574 r = 0.654, p<0.0001). This correlation was similar to that observed in Z536 (r = 0.518, p<0.0001) (Fig 5B). The two control strains Z537 and Z555 displayed exclusive *invA*_HA_ and *gfp* expression, respectively. However, a few cells displayed false positive signals. This was most likely attributable to noise or to slight cross-contaminations during the handling procedures of the staining protocol.

By and large, all strains showed equivalent levels of T1^+^ cells, irrespective of whether we used *gfp* or *invA*_HA_ as transcriptional reporter. Only the *gfp* expression under the control of the P*prgH* promotor showed ≈2-fold differences between the different strains, as the strain Z555 (only one reporter, i.e. P*prgH-gfp*) expressed a higher fraction of GFP^+^ cells than the strain Z536 (two reporters, i.e. P*prgH-gfp* and P*sicA-invA*_HA_) (Fig 5C). However, the average GFP fluorescence intensity was quite similar between Z555 and Z536 (Fig 5B). It remains unclear whether the 2-fold difference in the fraction of *gfp*-expressing cells might be attributable to the presence of two reporters in the same cell. However, this difference was much smaller when focusing on the InvA_HA_ expression (Fig 5C, bottom panel).

In conclusion, we could see similar correlation patterns in the strains harboring the different reporter constructs. Our data confirmed that InvA_HA_ can be used as reporter system to visualize gene expression and that this system can be used in combination with the *gfp* reporters.

## Discussion

Here, we established a novel system for the analysis of *S*. Tm *wbaP* gene expression at the single cell level. This strategy employs the epitope-tagged autotransporter invasin (InvA_HA_). Most previous reports studying the expression of individual promoters of interest within single cells have used fluorophores as reporter system [2, 22, 37, 43]. Using strains expressing *gfp* and *invA*_HA_ reporters from the same promoter (P*sicA*, Fig 5) or from two SPI-1 promoters (P*prgH* and P*sicA*, Fig 3), which were thought to be co-regulated, we could verify the suitability of this approach.

In theory, autotransporters like InvA may therefore provide distinct advantages when compared to *gfp*-based reporters. This is particularly relevant for cases when more than one promoter has to be analyzed per cell. First, it might offer a strategy to circumvent problems associated with the different folding/maturation half-lives observed with different fluorescent protein variants. Current approaches using different fluorophores for expression analysis within a single cell [2] impair accuracy due to different folding or maturation times. In future applications, it is conceivable that establishing a set of InvA_HA_ variants with equivalent maturation dynamics may allow such multi-gene expression analysis if one inserts multiple *invA* genes, harboring different epitope tags, e.g. *invA*_HA_ for P*prgH* and *invA*_Strep_ for P*sicA* expression analysis. Thus, it will be an important task for future work to establish the folding/maturation kinetics of the InvA_HA_ reporter (or similar reporters with longer extracellular extensions; see below) and verify that the kinetics are not affected by exchanging the HA epitope for other epitope members.

Clearly, there are important limitations that would have to be overcome before one can generally apply autotransporter reporters. It should be pointed out that the biological activity of wt autotransporters can have profound effects on pathogen-host interactions [24]. Thus, for extending the use of autotransporters to infection assays, it will be necessary to disrupt the host-cell binding. It seems conceivable that this can be achieved by introducing the epitope tag into the binding domain that is exposed on the bacterial surface. However, this would need to be addressed in future work.

Our current InvA_HA_ reporter system is limited to O-antigen-deficient *S*. Tm strains, as the full-length LPS layer prevents antibody binding of the HA epitope (Fig 1B). The same was true when we used an alternative reporter, i.e. the *E*. *coli* intimin carrying a Strep-tag reporter at its C-terminus (S2 Fig, [32]). Presumably, this is attributable to shielding by the long *S*. Tm O-antigen that includes up to 100 repeating units and has been estimated to form a 100 nm thick layer [44]. This may shield not only the HA-epitope of the InvA_HA_ reporter, but also the C-terminally located Strep-tag of intimin. In fact, changes in O-antigen layer thickness in *Shigella* spp. were reported to have dramatic effects on host cell invasion by shielding (or exposing) the tip of the invasion-mediating type three secretion system [45]. Thus, for designing autotransporter reporters, one may have to identify autotransporters that match the LPS O-sidechain length of the respective bacterium. In our pilot study, we have bypassed this problem by using an O-antigen deficient *wbaP* strain background. However, for many applications, the O-antigen-deficient background is not optimal, including our studies of T1 expression in wt *S*. Tm (Fig 2).

In previous reports analyzing the role of the O-antigen in *S*. Tm, an increased susceptibility to complement and antimicrobial peptides as well as a reduced colonization of mice was demonstrated in strains lacking the O-antigen [36, 46]. Moreover, these mutants show reduced swimming motility and an altered invasion efficiency in HeLa tissue culture models [36]. Thus, the O-antigen-deficient background is not very well suited for *in vivo* studies. In order to circumvent this problem, one would probably have to employ autotransporters that extend beyond the O-side chain and add tags to the surface-exposed moiety. This should alleviate the technical problems arising from the use of the O-antigen-deficient strain.

*S*. Tm is exquisitely sensitive to the expression of T1. The reduced growth rate of T1^+^ *S*. Tm cells is attributable (at least in part) to the metabolic costs associated with the expression of the T1 apparatus and the large pools of the pre-formed effector proteins [3, 16, 19]. It is known that the co-expression of T1 with a *gfp* reporter further enhances these costs. Thus, monitoring T1 expression represents a highly sensitive system to probe the "burden" imposed by any reporter construct. It is interesting to note that the *invA*_HA_ and the *gfp* reporters yielded equivalent results (one possible exception, Fig 5; Z536 vs Z555). This may suggest that the *invA*_HA_ reporter perturbs the system no more (or less) than the *gfp* reporter. As all reporters can potentially affect the system under scrutiny, one should carry out suitable control experiments to identify the most suitable system. Our *invA*_HA_ reporter thus expands the toolbox that one can choose from. Altogether, our data suggests that InvA_HA_ reporters should be quite well suited to replace *gfp* reporters for applications like the parallel analysis of several reporters of bacterial cells featuring a particular expression pattern, e.g. by MACS sorting.

**Fig 6:**
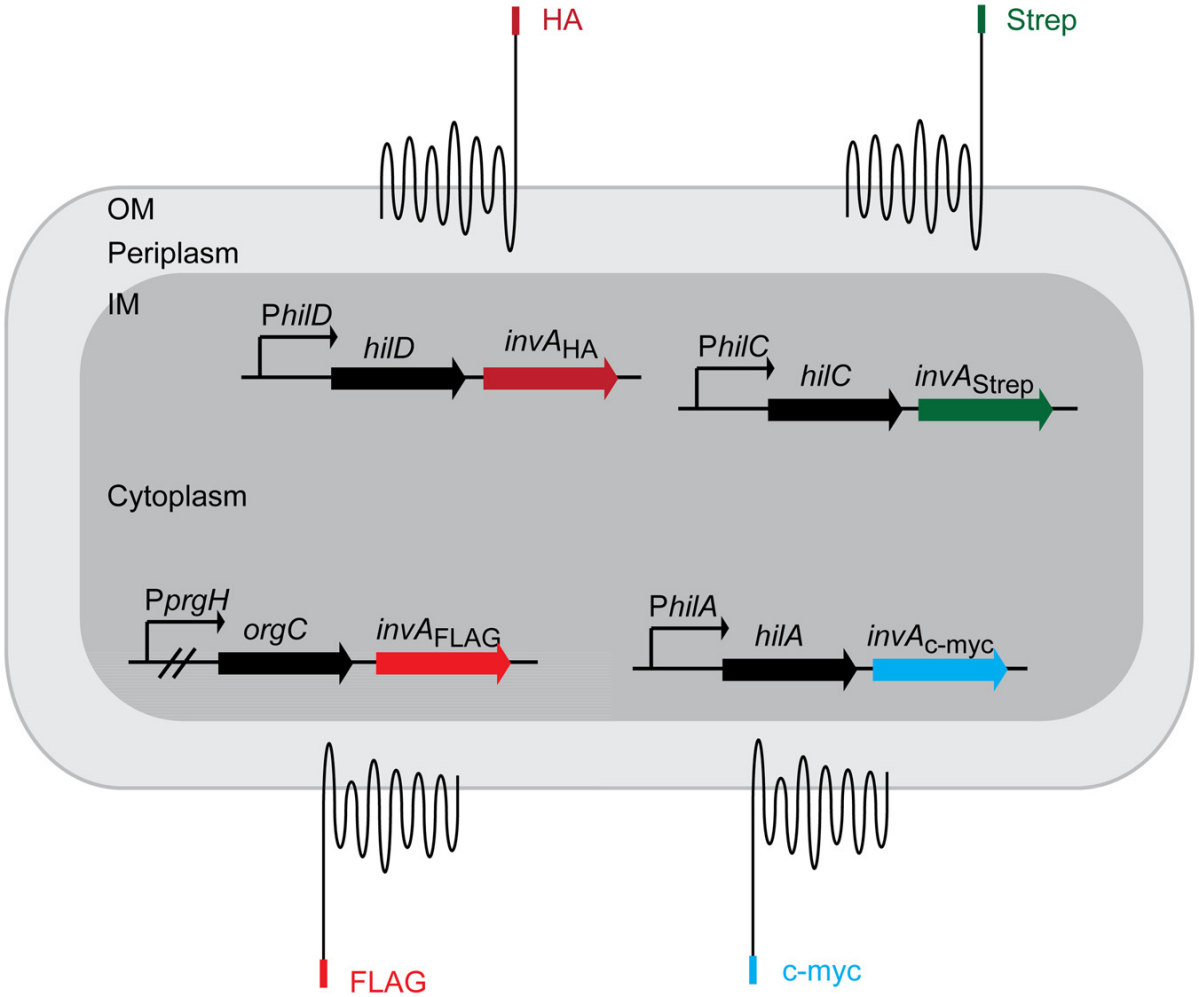
Potential extension to a multidimensional *invA*-based reporter system. Scheme of an *invA*-based reporter system employing four differentially tagged invasin autotransporters carrying epitopes inserted into the C-terminal region of the extracellular passenger domain. Surface staining for the epitope tags HA, Strep, c-myc, FLAG epitopes would thereby allow monitoring the intracellular expression of *hilD*, *hilC*, *hilA* and *prgH* by individual *S*. Tm cells (epitope tags and the corresponding genes are indicated by the same color). OM = outer membrane, IM = inner membrane.

In *S*. Tm, phenotypic diversity in the expression of the T1 virulence factor is well known [3, 16, 19, 22, 37, 43] and the core regulation factors controlling and fine-tuning this system are established (for reviews see [20, 21]. However, the exact mechanism behind the bistable behavior of the T1 system remains unknown. Analyzing the dynamics of the multiple transcription factors controlling the T1 regulatory cascade (e.g. HilD, HilC, HilA) within single cells would allow a deeper analysis of the fundamental organization of the complex regulatory cascade. An InvA system combining multiple *invA* genes (or similar reporters that allow surface detection even in the presence of wt LPS) with different epitope tags located at the outmost C-terminal region may provide a pioneering system for accurate assessment of T1 regulatory circuits (Fig 6). This would give further insights into the mechanisms behind the phenotypic diversity of the *S*. Tm T1 expression. We propose that the *invA*_HA_ reporter system, or variants thereof, can be used in a similar fashion for multi-dimensional gene expression analysis in Gram-negative bacteria at the single cell level.

## Supporting Information

S1 Fig Comparison of InvA_HA_ induction at the single-cell level by anhydrotetracycline- and arabinose-inducible plasmids. SKI-12 carrying either a tetracycline-inducible or an arabinose-inducible *invA*_HA_ cassette were cultured for 4 h in LB and exposed to the indicated concentrations of inducer. Living cells were stained using a HA-specific antibody, before they were analyzed by flow cytometry. Induction of InvA_HA_ expression was performed either by addition of AHTC or arabinose at the indicated concentrations. DH5α pInvA_HA_ was used as positive control for InvA_HA_ induction. Four to six measurements were performed in two independent experiments.
(TIF)

S2 Fig Surface staining of C-terminally Strep-tagged intimin. Flow cytometric analysis of the LPS-proficient SB300 strain, harboring either the wild type (Int wt) or Strep-tagged intimin (Int wt-Strep; [32]). The empty vector control pASK-IBA2 served as negative control for staining. The LPS-deficient strain SKI-12, carrying the Strep-tagged Intimin (SKI-12 Int wt-Strep) served as positive control for Strep-tag staining. Strep-tag expression was induced by addition of 200 ng/ml AHTC.
(TIF)
