# Supplementary figures and images for: Epitope-Tagged Autotransporters as Single-Cell Reporters for Gene Expression by a Salmonella Typhimurium wbaP Mutant
Source: PLoS One. 2016 May 5;11(5):e0154828. doi: 10.1371/journal.pone.0154828 (PMC4858243; doi:10.1371/journal.pone.0154828)

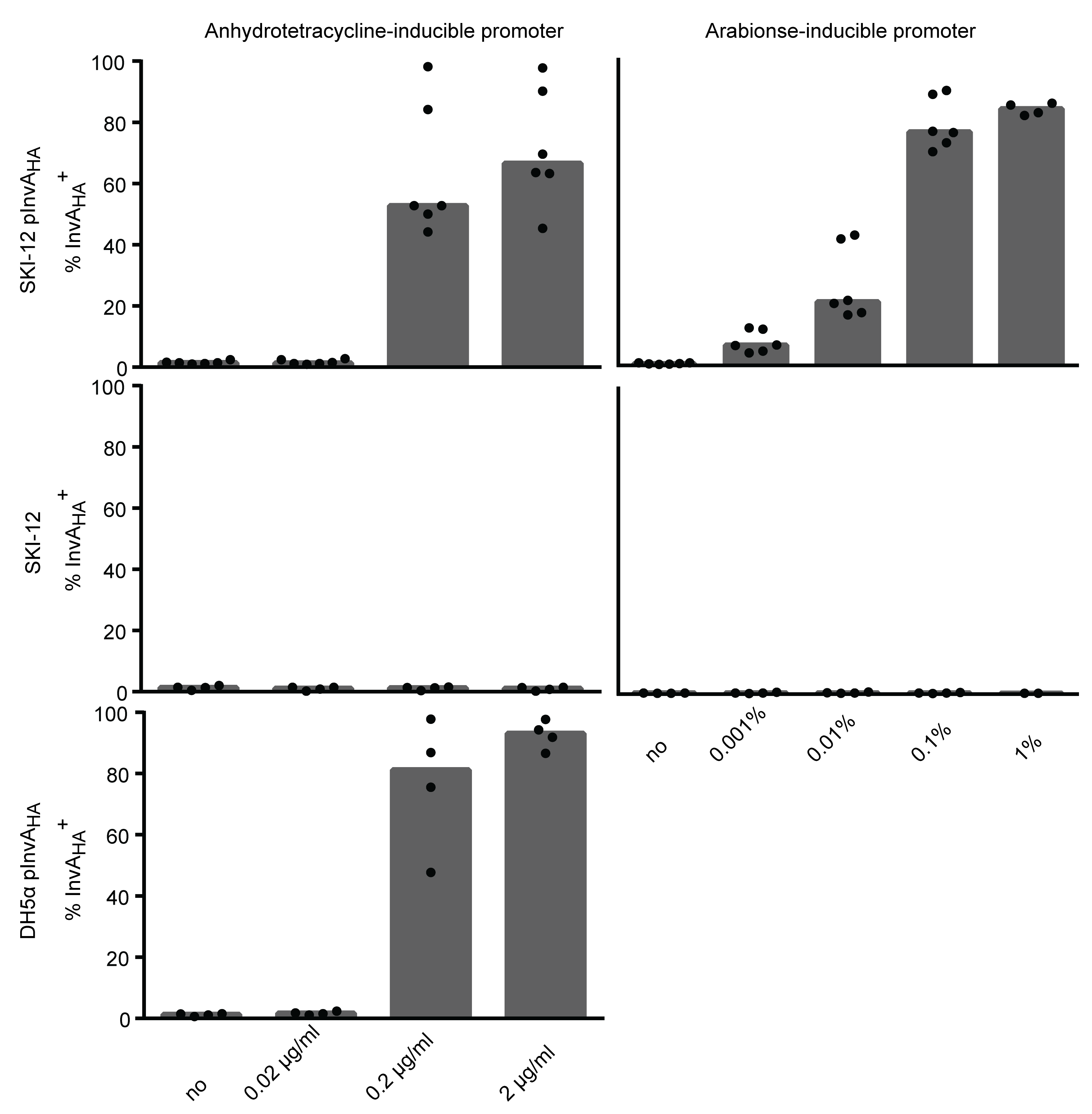

Supplement: S1 Fig — SKI-12 carrying either a tetracycline-inducible or an arabinose-inducible invAHA cassette were cultured for 4 h in LB and exposed to the indicated concentrations of inducer. Living cells were stained using a HA-specific antibody, before they were analyzed by flow cytometry. Induction of InvAHA expression was performed either by addition of AHTC or arabinose at the indicated concentrations. DH5α pInvAHA was used as positive control for InvAHA induction. Four to six measurements were performed in two independent experiments. (TIF) [file pone.0154828.s001.tif]

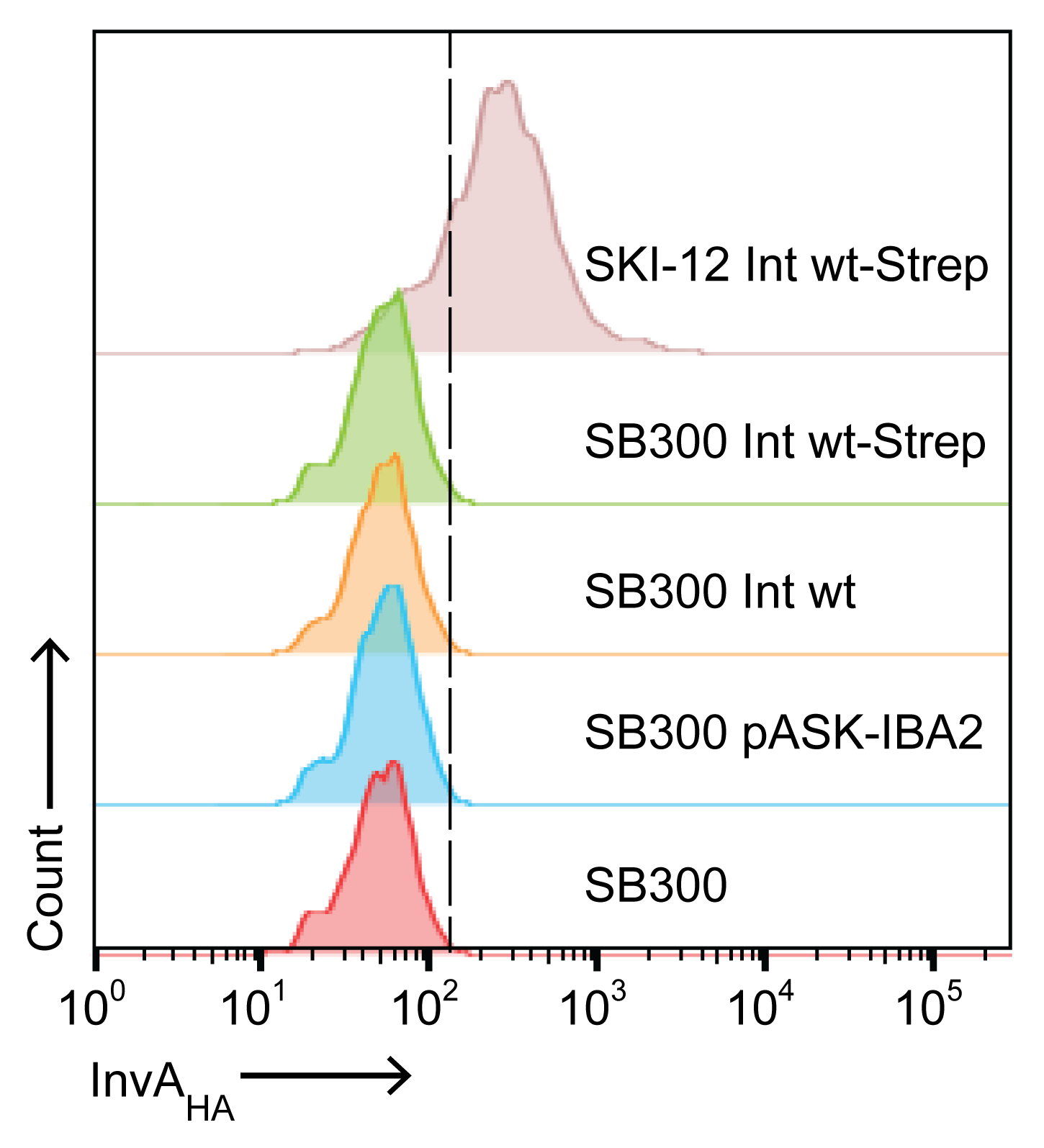

Supplement: S2 Fig — Flow cytometric analysis of the LPS-proficient SB300 strain, harboring either the wild type (Int wt) or Strep-tagged intimin (Int wt-Strep; [32]). The empty vector control pASK-IBA2 served as negative control for staining. The LPS-deficient strain SKI-12, carrying the Strep-tagged Intimin (SKI-12 Int wt-Strep) served as positive control for Strep-tag staining. Strep-tag expression was induced by addition of 200 ng/ml AHTC. (TIF) [file pone.0154828.s002.tif]
